# Sequencing and Analysis of the Genome of the Filamentous Fungus *Penicillium verrucosum* BFE808, Which Produces the Mycotoxins Citrinin and Ochratoxin

**DOI:** 10.1128/MRA.00953-18

**Published:** 2018-10-11

**Authors:** Markus Schmidt-Heydt, Dominic Stoll, Rolf Geisen

**Affiliations:** a Department of Safety and Quality of Fruits and Vegetables, Max Rubner-Institut, Federal Research Institut for Nutrition and Food, Karlsruhe, Germany; Vanderbilt University

## Abstract

Penicillium verrucosum is a filamentous ascomycete that occurs worldwide. Various cereals and the products thereof are the main habitats of this fungal species, where it produces the mycotoxins ochratoxin and citrinin. Here, we report the first draft genome sequence of P. verrucosum strain BFE808, isolated from wheat kernels.

## Abstract (precis)

Penicillium verrucosum is a filamentous ascomycete that occurs worldwide. Various cereals and the products thereof are the main habitats of this fungal species, where it produces the mycotoxins ochratoxin and citrinin.

## ANNOUNCEMENT

Penicillium verrucosum was first isolated in Belgium and described by Dierckx in 1901 (http://www.mycobank.org/BioloMICS.aspx?TableKey=14682616000000063&Rec=14971&Fields=All). Initially, this species was placed in synonymy with Penicillium viridicatum, but it was later revised as a separate species (1–3). Both P. verrucosum and P. viridicatum are able to produce the mycotoxin citrinin, but P. verrucosum is also a producer of the mycotoxin ochratoxin (3, 4). Nowadays, P. verrucosum belongs to the series *Verrucosa* within the section *Fasciculata* together with Penicillium nordicum and Penicillium thymicola (5, 6). P. verrucosum is psychrotolerant and occurs mainly in moderate-temperature regions, such as Northern Europe, North America, Canada, and parts of South America. The strain P. verrucosum BFE808, whose genome sequence is announced here, was isolated from wheat kernels and produces the mycotoxins ochratoxin and citrinin (4), which both have detrimental effects on the kidneys, liver, and immune system (7).

Genome sequencing was done on the MiSeq platform (Illumina, USA) after P. verrucosum BFE808 was grown for 7 days at 25°C in yeast extract-sucrose (YES) broth (20 g/liter yeast extract, 150 g/liter saccharose). DNA was extracted using the NucleoSpin Plant II kit (Macherey-Nagel, Germany) and was quantified and quality checked using NanoDrop 1000 (VWR International, Germany) and the Qubit 3.0 photometer, respectively. The sequencing library was created using the Illumina Nextera kit and quality checked using the Experion DNA 1K analysis kit (Bio-Rad Laboratories, Germany). The raw data were processed with Blast2GO Pro V5.2 using the FASTQ processing toolkit. *De novo* assembling was done using SeqMan NGen V15 (DNASTAR, USA) with the following parameters: quality end trim, yes; k-mer size, 21 nucleotides (nt); match percentage,  >93%; realign reads, yes; and remove small contigs <200 nt, yes. Mitochondrial sequences were removed. The genome assembly was 30,246,699 bp long with 60× coverage containing 1,766 genomic contigs; the *N*_50_ value was 36,626 kb, and the G+C content was 48.1%. Prediction of biosynthesis gene clusters (BGCs) was carried out using antiSMASH fungal version V3.0 with the cluster finder algorithm for BGC border prediction (8) and the following parameters: cluster size of coding DNA sequences (CDS), >5; Pfam domains, >5; and cluster probability, >60%. Of the 48 BGCs which have been predicted, 15 could be categorized as type 1 polyketide synthase (T1 PKS) clusters, 7 as nonribosomal peptide synthase (NRPS) type clusters, 4 as PKS-NRPS hybrid clusters, 1 as an indole-NRPS hybrid cluster, 2 as fatty acid type clusters, 4 as terpene type clusters, and 2 as indole type clusters; 13 putative BGCs were not further specified. Genome annotation based on Aspergillus nidulans as the closest related species with available annotation data was done using Blast2GO Pro V5.2 (9), resulting in 10,078 identified genes.

Within the genome sequence of P. verrucosum BFE808, the biosynthesis gene clusters for ochratoxin and citrinin were identified; these are two important mycotoxins whose legal limits in foods are regulated by federal law. The ochratoxin cluster shows homology to the ochratoxin biosynthesis cluster of Aspergillus westerdijkiae (10), whereas the citrinin cluster is similar to that of Monascus purpureus (11). Moreover, we also found homologues of the ochratoxin cluster in other penicillia (12, 13). Future analyses of the genome sequence of this highly important mycotoxin-producing fungal species will give a deeper insight into secondary metabolite biosynthesis of P. verrucosum in comparison to other ochratoxin- and citrinin-producing fungi.

### Data availability.

This whole-genome shotgun project of P. verrucosum has been deposited at NCBI GenBank under the accession number LAKW00000000. Raw sequence reads can be found under the SRA accession number SRR7851424.
